# An Immunohistochemical Analysis to Validate the Rationale behind the Enhanced Immunogenicity of D-Ribosylated Low Density Lipo-Protein

**DOI:** 10.1371/journal.pone.0113144

**Published:** 2014-11-13

**Authors:** Firoz Akhter, M. Salman Khan, Sarika Singh, Saheem Ahmad

**Affiliations:** 1 Department of Bio-Engineering, Integral University, Lucknow, India; 2 Department of Bio-Sciences, Integral University, Lucknow, India; 3 Department Toxicology, Central Drug Research Institute (CDRI), Lucknow, India; University of Leicester, United Kingdom

## Abstract

Advanced glycation end products (AGEs) are thought to contribute to the abnormal lipoprotein profiles and increased risk of cardiovascular disease in patients with diabetes and renal failure. D-ribose is one of the naturally occurring pentose monosaccharide present in all living cells and is a key component of numerous biomolecules involved in many important metabolic pathways. Formation of D-ribose derived glycated low density lipoprotein (LDL) has been previously demonstrated but no studies have been performed to assess the immune complex deposition in the kidney of rabbits immunized with glycated LDL. In this study, LDL was glycated with D-ribose, and it was further used as an immunogen for immunizing NZW female rabbits. The results showed that female rabbits immunized with D-ribose modified LDL induced antibodies as detected by direct binding and competitive ELISA. The modified LDL was found to be highly immunogenic eliciting high titer immunogen-specific antibodies, while the native forms were moderately immunogenic. The induced antibodies from modified LDL exhibited wide range of heterogeneity in recognizing various proteins and amino acids conformers. Furthermore, our histopathological results illustrated the deposits of immune complex in glomerular basement membrane in rabbits immunized with D-ribose-LDL.

## Introduction

Non-enzymatic glycation of proteins is a post-translational modification process [1], leading to the formation of fructosamine [2] and advanced glycation end products (AGEs) [3], [4]. It has also been reported that there is the generation of oxygen free radicals in the formation of early and advanced glycation end-products [5]. Basically, glycation is a condensation of free carbonyl group of reducing sugars with a free amino group of DNA, proteins and LDL macromolecules. The reaction proceeds by nucleophilic addition reaction followed by dehydration, cyclization and rearrangement to form AGEs. The role of glucose in the glycation of proteins has been widely studied however the role of other reducing monosaccharides such as D-ribose in glycation and their resulting effects on animal model has received much less attention. AGEs are heterogeneous class of compounds containing a mixture of cyclic and open chain structures out of which large numbers of AGEs are not determined till date. However, few AGEs which are known like pentosidine, carboxymethyl-lysine (CML), carboxyethyl-lysine (CEL), fructosyl-lysine (FL), which plays an important role in atherosclerosis and nephropathy. The lipoprotein glycation process occurs both on the apoprotein B (apoB) and on phospholipid components of LDL. It leads both functional alterations in LDL clearance and increased susceptibility to oxidative modifications [6]. In addition, oxidative stress and other conditions causing oxidation of LDL stimulate the synthesis of nuclear polymers of adenosine diphosphate ribose (ADP-ribose), which in turn can release monomeric ADP-ribose. The bio-availability of D-ribose makes this carbonyl species quite reactive and damaging, therefore having direct implication in diabetes.

There are ample evidences which indicate the role of lipoprotein glycation in the inflammatory reactions in the vessel wall [7]. Various oxidative stresses of different proteins may be involved in proinflammatory immune mechanisms. These may involve innate immune signals, Toll-like receptors [8] and adaptive immunity signals which are both cell-mediated [9] and antibody-mediated [10]. There are ample of evidences supporting the pathogenic role of humoral response to modified lipoproteins [11]–[14]. This is mainly due to the fact that modified LDL and its corresponding antibodies form modified LDL immune complexes (mLDL-IC), which are able to activate phagocytic cells through engagement of Fc receptors [14]. Engagement of Fc receptors by mLDL-IC is particularly significant because it delivers stronger activating signals to phagocytic cells than the engagement of scavenger receptors by modified LDL [11]. Native LDL was found to be moderately immunogenic in experimental animals, while, modified form of LDL was found to be highly immunogenic [15].

This study was aimed to see the immunogenicity of native and glycated LDL. The cross reactivity experiments were performed to check the specificity of the raised antibodies using various glycated and non-glycated inhibitors including poly amino acids. Moreover, the immuno-histochemistry was performed to see the immune complex deposition in the kidney of immunized female rabbits.

## Materials and Methods

### Ethics statement

The immunization work was approved by Institutional Animal Ethical Committee of Integral University, Lucknow, India by approval No: IU/Biotech/Project/CPCSEA/12/20. The animals were treated with utmost care and treatments were done with minimum pain. The *in vivo* experiments on rabbits were performed according to the ARRIVE guidelines. After the end of experiment the rabbits were sacrificed by the over dosage of thiopentone sodium anesthesia by intra venous mode of injection (≥150 mg/kg).

### Materials

D-ribose, Tris-HCl, EDTA, PTA, TBA, Protein A-Agarose column were obtained from Sigma, St. Louis, MO. Polystyrene plates obtained from Nunc (Roskilde, Denmark), HSA, Hb, IgG, Poly-L-lysine, Poly-L-arginine, Poly-L-histidine were obtained from MP Biomedicals and LDL, Anti rabbit IgG, pNPP were purchased from Calbiochem. All other chemicals used in this study were of highest analytical grade available in the country.

### Glycation of LDL

LDL was modified by using different concentrations of D-ribose [16]. LDL (62.5 µg/ml) was thoroughly mixed with 80 mM of ribose sugar in 100 mM phosphate buffer saline (PBS), pH 7.4 and incubated at 37°C for three weeks, followed by extensive dialysis against phosphate buffer saline to remove unbound constituents. LDL alone was served as control [17].

Various proteins like HSA, Hb, IgG and Poly amino acids like Poly-L-lysine, Poly-L-arginine and Poly-L-histidine were also modified by using D-ribose with same concentration and with the same procedure [17].

### Measurement of superoxide anion

To detect the presence of O2˙– anion in the LDL glycation mixture, a cytochrome-c reduction assay was performed. The reaction mixture contained D-ribose (80 mM) and 100 µM cytochrome-c in 20 mM phosphate buffer (pH 7.4). The reduction rate was determined as the increase in absorbance at 550 nm for 10 min at room temperature. Absorbance was taken at one min intervals [18].

### Measurement of hydroxyl radical

Detection of hydroxyl radicals was carried out by measuring thiobarbituric acid (TBA) reactive 2-deoxy-D-ribose oxidation products. Reaction mixtures containing 80 mM D-ribose, and LDL (62.5 µg/ml) was incubated at 37°C for two weeks. The degradation of 2-deoxy-D-ribose was measured by adding l mL of 2.8% (w/v) trichloroacetic acid, 1 mL of 1% (w/v) thiobarbituric acid followed by heating at 100°C for 10 min. After cooling the absorbance was read at 532 nm [19].

### Immunization Schedule

LDL (25 µg), its D-ribose modified counterpart and D-ribose (25 µg) were emulsified with complete Freund’s adjuvant. The complex and 500 µl CFA were injected intramuscularly in the hind leg muscles of rabbits (three animals in each group). Subsequent injections were given in incomplete Freund’s adjuvant until the period of study. Weekly injections of antigen (LDL/D-ribose modified LDL/D-ribose) were given for seven weeks and thus each animal received a total of 175 µg antigen during the course of immunization. One week after the last dose of immunogen, blood was collected and put it at 37°C for 2–3 hours. The serum separated and decomplemented by heating at 56°C for 30 min on the water bath. Preimmune blood was collected prior to immunization. The serum was stored in small aliquots at −80°C with sodium azide (0.1%) as preservative [20].

### Estimation of Conjugate diene (CD) and Thiobarbituric acid reducing substance (TBARS) in plasma

The blood from each NZW female rabbit in a given group was collected in heparinized tubes, for 2–3 h, mixed gently by inversion 2–3 times and incubated at 40°C. Plasma was separated from blood by centrifugation at 2,500 rpm for 30 min, aliquoted and stored at –20°C.

For the extraction of Lipid contents from plasma, the method of Folch *et al.* [21] was employed. One volume of plasma was mixed with 5.0 volume of chloroform: methanol (2∶1), followed by centrifugation at 1,000 rpm for 5 min to separate the phases. Most of the upper layer was removed; and 3.0 ml of the lower chloroform layer was recovered. The chloroform layer was placed in a test tube and incubated at 45°C till dryness. For the determination of conjugated diene (CD) in plasma, corresponding lipid residues were dissolved in 1.5 ml of cyclohexane and the absorbance was recorded at 234 nm against a cyclohexane blank in a Beckman DU 640 spectrophotometer. The concentration of conjugated diene formation was calculated by using a molar extinction coefficient of 2.52×l 0^4^ M^−l^cm^−l^.

Lipid peroxide conte0nts in plasma were assayed by the method of Yagi [22]. Plasma (25 µl) was mixed with 4.0 ml of 0.083 N H_2_SO_4_ followed by the addition of 0.5 ml of 10% phosphotungstic acid. The samples were mixed and incubated for 5 min at room temperature and then centrifuged at 3,000 rpm for 10 min. The supernatant was discarded and the sediment was mixed with 2.0 ml of 0.083 N H_2_SO_4_ and 0.3 ml of 10% phosphotungstic acid. The mixture was centrifuged at 3,000 rpm for 10 min, the sediment was suspended in 4.0 ml of water and 1.0 ml of TBA reagent (a mixture containing equal volumes of 0.67% aqueous TBA solution and glacial acetic acid) was added. The reaction mixture was heated for 60 min at 95°C, cooled and the tubes were centrifuged at 3,000 rpm for 10 min. The absorbance of the supernatant was determined at 532 nm against a reagent blank in an Eppendorf Bio Spectrometer. The concentration of MDA was calculated by using a standard malondialdehyde [23].

### Enzyme linked immunosorbent assay (ELISA)

ELISA was performed on polystyrene plates [24] with slight modification. Polystyrene polysorp immunoplates were coated with 100 µl of the D-ribose, native or glycated LDL (10 µg/ml) in 0.05 mol/l carbonate–bicarbonate buffer, pH 9.6. The plates were incubated for 2 hours at 37°C and overnight at 4°C. Each sample was coated in duplicate and half of the plate, devoid of antigen coating, served as control. Unbound antigen was washed three times with TBS-T (20 mmol/l Tris, 150 mmol/l NaCl, pH 7.4 containing 0.05% Tween-20), and unoccupied sites were blocked with 2% fat-free skimmed milk in TBS (10 mmol/l Tris, 150 mmol/l NaCl, pH 7.4) for 6 hours at 37°C. After incubation, the plates were washed again three times with TBS-T. Test serum was added to antigen-coated wells and reincubated for 2 hours at 37°C and overnight at 4°C. Bound antibodies were assayed with anti-rabbit IgG-alkaline phosphatase conjugate using *p*-nitrophenyl phosphate (pNPP) as substrate. Absorbance (A) of each well was monitored at 410 nm on an automatic microplate reader, and the mean of duplicate readings for each sample was recorded. Results have been expressed as a mean of A_test_-A_control_.

### Competitive ELISA/Cross reactivity

The antigen-binding specificity of the antibodies was evaluated by competitive ELISA and cross reactivity [25]. In direct binding ELISA D-ribose, N-LDL and G-LDL (0–20 µg/ml) were mixed with constant amount of induced respective sera. In cross reactivity varying amounts of inhibitors like native and D-ribose modified proteins (LDL, HSA, Hb, IgG) and amino acids (Poly-L-lysine, Poly-L-arginine, Poly-L-histidine) (0–20 µg/ml) were mixed with a constant amount of IgG. The mixture was incubated at room temperature for 2 hours and overnight at 4°C. The immune complex thus formed was added to the wells, in place of serum. The remaining steps were same as in direct binding ELISA. Percent inhibition was calculated using the following formula:

Percent Inhibition = 1−(A_inhibited_/A_uninhibited_)×100.

In order to perform the cross reactivity, firstly the IgG antibodies were isolated using protein-A-Agarose column, based on affinity chromatography.

### Purification of Antibodies

Immunoglobulin G (IgG) was affinity purified from preimmune and immune sera on a protein A-Agarose column [26]. Serum (0.5 ml) diluted with equal volume of PBS (pH 7.4), was applied to the column (0.9×15 cm) pre-equilibrated with above buffer. The wash through was recycled 2–3 times. Unbound IgG was removed by extensive washing with PBS (pH 7.4). The bound IgG was eluted with 0.58% acetic acid in 0.85% sodium chloride [27]. Three milliliter fractions were collected in a measuring cylinder already containing 1 ml of 1 M Tris–HCl, pH 8.5, and absorbance was recorded. The IgG concentration was determined considering 1.4 OD_280_ = 1.0 mg mammalian IgG/ml. The isolated IgG was then dialyzed against PBS (pH 7.4) and stored at −80°C.

### Histopathological study of Kidney Sections

#### Light Microscopic analysis

Slices of kidney from rabbits immunized with native and modified LDL was fixed in 10% formalin, dehydrated with increasing strength of ethanol, cleared in xylene, and wax impregnated. Paraffin blocks of the kidney pieces were fixed to block holder to cut 5-µm thick sections. Sections were dewaxed with xylene and hydrated using descending grades of alcohol. Hematoxylin (nuclei stain) and eosin (cytoplasmic stain) were used for staining. Again, the sections were dehydrated with ascending grades of alcohol and cleared with xylene before finally mounting in a mixture of distyrene, a plasticizer, and xylene (DPX) for microscopic observation [28].

#### Immunofluorescence analysis

5 µm thick sections were cut with the help of a cryostat at −30°C. Sections were fixed with acetone on glass slides, incubated with anti-rabbit IgG fluorescein isothiocyanate conjugate (FITC) obtained from Sigma (St. Louis, MO) for 30 min. After three wash with phosphate buffer saline (PBS, pH 7.4), the sections were mounted with 50% glycerol and viewed under fluorescent microscope [28].

## Results

### Modification of LDL by D-ribose

We previously demonstrated that *in* *vitro* treatment of commercially available LDL with D-ribose results in biophysical and biochemical alterations in LDL to form LDL-AGEs [16].

### Estimation of superoxide anion

Superoxide generation in the D-ribose modified human LDL was quantitated by a cytochrome c reduction experiment. During incubation of D-ribose with LDL, the formation of superoxide anion was gradually increased in time dependent manner. The incubation of D-ribose with LDL produced 30.5 nmol O2˙– ml^−1^ h^−1^ compared to 2.34 nmol O2˙– ml^–1^ h^−1^ with D-ribose alone. Superoxide dismutase (SOD) inhibited the superoxide radical generation. Upon, increasing concentration of SOD, the superoxide generation was gradually decreased.

### Estimation of hydroxyl radicals

Incubation of 2-deoxy-D-ribose with D-ribose produced 14.2 nmol TBARS ml^−1^ however; reaction of LDL with D-ribose in the presence of Fe^3+^ enhanced it to 22.1 nmol TBARS ml^−1^. Radical scavengers like mannitol, catalase and a metal ion chelator, desferrioxamine significantly inhibited the production of TBARS. TBARS generation was inhibited up to 58.3% when mannitol was used as a scavenger. However, catalase and desferrioxamine inhibited TBARS up to 52.4 and 60.2% respectively. The result suggests that D-ribose-mediated hydroxyl radical generation may be caused by traces of transition metals. Furthermore, it is also being suggested that the redox reactions of iron may facilitate the generation of hydroxyl radical by reaction of LDL and D-Ribose.

### Estimation of CD and TBARS in plasma

CD in plasma was significantly increased in D-ribose glycated LDL induced plasma of NZW female rabbit in comparison preimmune plasma while moderately increased in D-ribose, CFA and N-LDL induced plasma (Table 1). TBARS in plasma was also significantly increased in D-ribose glycated LDL induced plasma in comparison to preimmune plasma while a very less or no change in D-ribose, CFA and N-LDL induced plasma (Table 1).

**Table 1:** Effect of CFA, D-ribose, N-LDL and G-LDL on plasma TBARS and Conjugated Diene (CD) in control and treated NZW female rabbits.

S.No.	Sera	Conjugated diene (CD)	TBARS
1.	Preimmune sera	15.15 nM±0.041 nM	0.48 µM±0.044 µM
2.	CFA immunized sera	22.5 nM±0.17 nM	0.044 µM±0.006 µM
3.	D-ribose immunized sera	27.22 nM±0.73 nM	0.09 µM±0.03 µM
4.	N-LDL immunized sera	27.38 nM±0.13 nM	0.112 µM±0.042 µM
5.	G-LDL immunized sera	54.76 nM±0.088 nM	3.85 µM±0.057 µM

Each data represents average of three experiments. The values represent the mean ± SD.

### Immunogenicity of LDL and its D-ribose-modified form

**Figure 1:**
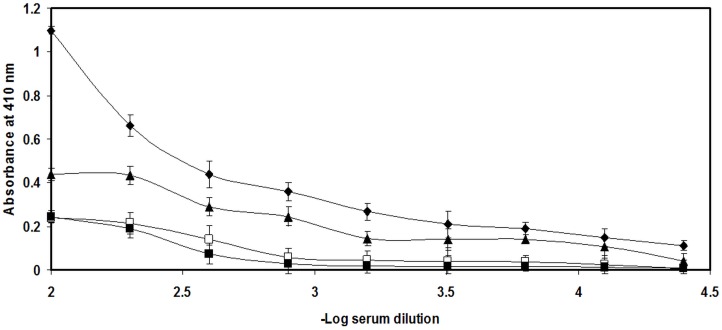
Level of induced antibodies against D-ribose-modified LDL. Direct binding ELISA of D-ribose antisera (□), N-LDL antisera (▴), G-LDL antisera (♦) and preimmune sera (▪). The microtiter wells were coated with D-ribose, N-LDL and G-LDL (10 µg/ml) in direct binding ELISA of D-ribose antisera, native LDL antisera and G-LDL antisera respectively. Each data represents average of three experiments. The values represent the mean ± SD.

In the present study, antigenicity of D-ribose, CFA, native and D-ribose-modified LDL was probed in New Zealand white (NZW) female rabbits. The result from direct binding ELISA D-ribose showed not significant binding, native LDL was moderately immunogenic eliciting average titer of antibodies, while its modified form i.e. glycated LDL was found to be much more immunogenic than its native form (Figure 1). CFA not showed significant binding with its induced sera (data not shown). Native LDL showed the titer value of >1∶3200 while D-ribose glycated LDL antiserum showed high titer antibodies (>1∶25,600) in direct binding ELISA. Binding of the preimmune serum was of low magnitude (Figure 1).

**Figure 2:**
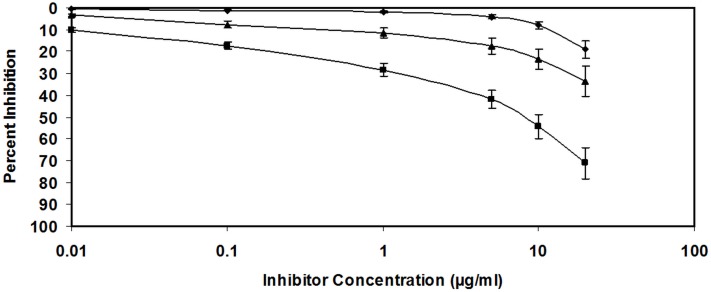
Inhibition of serum antibodies against N-LDL and G-LDL binding by D-ribose (♦), N-LDL (▴) and G-LDL (▪) respectively. The microtiter plate was coated with D-ribose, N-LDL and G-LDL (10 µg/ml) respectively. Each data represents average of three experiments. The values represent the mean ± SD.

Furthermore, in the competitive inhibition ELISA we used D-ribose glycated LDL antiserum to explore its specificity against the immunogen i.e. D-ribose glycated LDL (Figure 2).

The purified IgG from protein A-Agarose column showed single homogenous band on denaturing SDS-PAGE (data not shown).

The IgG antibodies exhibited wide range of heterogeneity in recognizing varied inhibitors that included native and glycated analogues of LDL, HSA, Hb, IgG, poly L-lysine, poly L-arginine and poly L-histidine. A maximum of 82.3% inhibition of the D-ribose-LDL-IgG antibody was observed with the immunogen, when it was used as an inhibitor (Figure 2). The antibody was highly specific for the immunogen as only 2.5 µg/mL D-ribose modified-LDL caused 50% inhibition in the antiserum activity (Table 2). The IgG antibody showed considerably low recognition for native LDL, which caused a maximum inhibition of only 33.4%. The inhibition of D-ribose-LDL-IgG antibody by various competitors is given in Table 2. IgG concentration was kept constant (40 µg/ml) in all experiments. The cross reactivity observed with various amino acids (poly L-lysine, poly L-arginine, poly L-histidine) and protein (HSA, Hb, IgG) was due to the recognition of antigenic determinants. Reports show that D-ribose induces maximum modification at arginine and lysine residue in modified LDL case (Table 2), which explains that the higher cross reactivity of antiserum with D-ribose-arginine and D-ribose-lysine. This result is corroborated with the previously published reports [29].

**Table 2:** Cross reaction of anti-D-ribose-modified LDL IgG.

S.No.	Inhibitors	Maximum % inhibitionAt 20 µg/ml	Concentration for 50%inhibition (µg/ml)	Percent relative affinity
**1.**	D-ribose-LDL	82.3±0.71	2.5±0.04	100±0.04
**2.**	Native LDL	33.4±0.15	–-	–-
**3.**	D-ribose-HSA	46.22±1.34	–-	–-
**4.**	Native HAS	32.15±1.03	–-	–-
**5.**	D-ribose-Hb	52.03±1.74	18.4±0.12	13.59±0.25
**6.**	Native Hb	24.21±0.78	–-	–-
**7.**	D-ribose-IgG	48.101±0.86	–-	–-
**8.**	Native IgG	23.17±0.23	–-	–-
**9.**	D-ribose-Poly-L-lysine	56.8±1.81	15.3±0.22	16.34±0.26
**10.**	Native Poly-L-lysine	27.27±0.32	–-	–-
**11.**	D-ribose-Poly-L-arginiine	58.312±1.32	13.7±0.11	18.25±0.28
**12.**	Native Poly-L- arginiine	18.27±0.21	–-	–-
**13.**	D-ribose-Poly-L-Histidine	39.78±0.42	–-	–-
**14.**	Native Poly-L- Histidine	26.022±0.32	–-	–-

Micro titer plates were coated with D-ribose-modified LDL (10.0 µg/mL). Each data represents average of three experiments. The values represent the mean ± SD.

**Figure 3:**
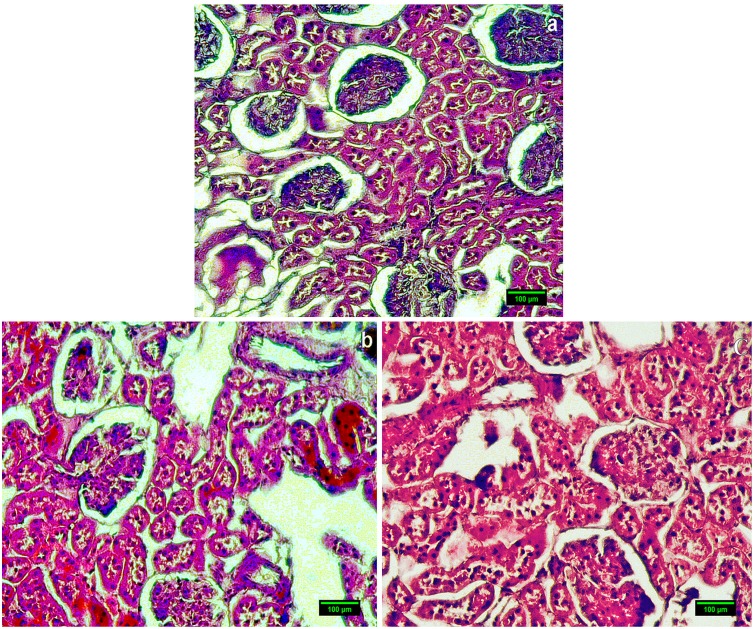
Kidney section from female rabbit immunized with native LDL showing normal morphology of glomeruli (a) (Magnification-60x); whereas, Kidney section from female rabbit immunized with D-ribose-modified LDL showing larger glomerular capillary tuft with increased number of nuclei showing proliferation of endothelium and mesenglial cells (b & c) (Magnification-60x). Scale bar 100 µm.

**Figure 4:**
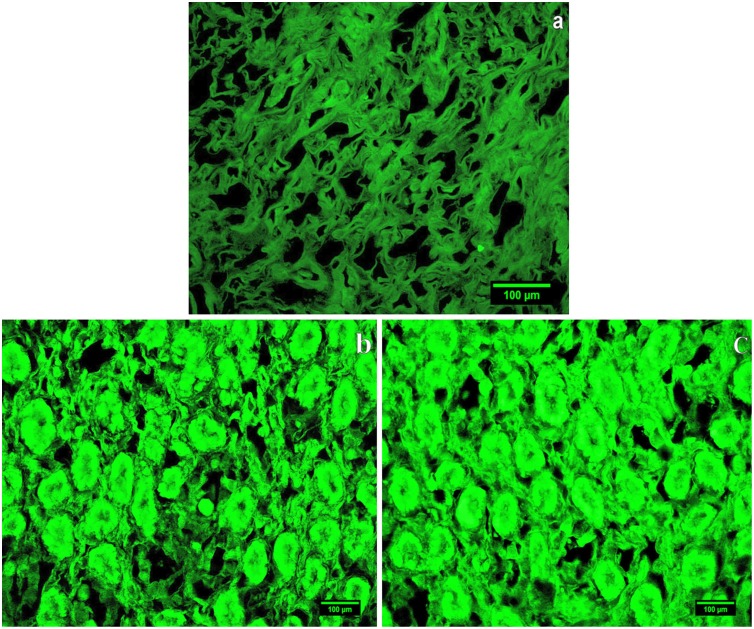
Immunofluorescence of kidney sections of rabbit immunized with native LDL (a); whereas Immunofluorescence of kidney sections of rabbit immunized with D-ribose-modified LDL showing immune complex deposition on GBM (b & c). Scale bar 100 µm.

In Histopathological study of NZW female rabbit’s immunized with native LDL showing normal morphology of glomeruli (Figure 3a) while female rabbit’s immunized with D-ribosylated LDL showing larger glomerular capillary tuft with increased number of nuclei (Figure 3b and 3c). In the fluorescence microscopic examination, low fluorescence signal was detected in FITC-labelled kidney sections of rabbits immunized with native LDL (Figure 4a) while D-ribosylated-LDL-immunized rabbit’s kidney section showing GBM having a series of intense fluorescence points (Figure 4b and 4c).

## Discussion

The LDL concentration used in these experiments for glycation is 62.5 µg/ml. The average level of LDL in normal individual is 1 mg/ml, However, the level of glycated LDL in diabetic subjects is 30 µg/ml. Therefore, to see the significant change in LDL macromolecule we used double the amount of glycated LDL in diabetic subjects which is 62.5 µg/ml.

Furthermore, a small spectrum of information like body weight, food consumption and illness were observed in phenotypic studies. In-life parameters we have monitored daily clinical observations, weekly body weights. Up to 3rd week of immunization there was no phenotypic changes were observed moreover no significant alterations were found in the total daily food consumption and weekly food consumption without any illness. We also observed that animals immunized with glycated LDL were more prone to reduction in body weight & food consumption as composed to native LDL.

Ribose is a naturally occurring pentose monosaccharide present in all living cells including the blood and is a key component of many important bio-molecules such as riboflavin, RNA and ATP [30]. Our study has shown that D-ribose causes structural perturbations in LDL molecule resulting in the generation of neo-antigenic epitopes which are recognized as non-self by the immune system, thus breaking the immune tolerance existing normally to self-antigens. Previously it has been shown that oxidized form of the LDL is highly immunogenic and shows potential recognition of auto-antibodies raised against modified LDL [31]. This is the first study in which we explored the immunogenicity of native and glycated LDL by D-ribose. Our results also point towards the possible involvement of this pentose sugar in the induction of antibody response in different diseases. Thus, it is reasonable to conclude that at present the exact pathophysiological role of glycation of apo B is still obscure and needs further study.

Vigorous humoral response in animals immunized with D-ribose-modified LDL suggests an alteration in the LDL structure, resulting in the generation of neo-epitopes, which are recognized as nonself by the immune system, leading to the robust production of antibodies.

The half-life of LDL in the circulation is in the order of 3–4 days. Some research groups experimentally showed in rabbits that *in* *vitro* glycated low density lipoprotein (glycated-LDL) was cleared at a slower rate than control LDL and thus stayed longer in the circulation (vascular mean residence time: 10 vs 8 h, p less than 0.001). The bodies mean residence time for glycated-LDL was 22 h vs 17 h for control LDL. In diabetic animals the catabolic parameters of both LDL preparations changed towards a faster clearance, the effect being greatest for glycated-LDL (total mean residence times of glycated-LDL pre-diabetic: 19 h, diabetic: 16 h; control LDL pre-diabetic and diabetic: 14 h). The difference in clearance between glycated and control LDL was thus strongly reduced. This suggests an increased activity of the non-receptor mediated pathway in diabetes mellitus, possibly co-responsible for an increased atherosclerotic risk [32].

Histological examination of kidney sections of rabbits immunized with native LDL showed a normal kidney morphology having normal glomerular, tubular, and vascular structure (Figure 3a). Whereas D-ribose-LDL immunized rabbits revealed hyper cellular and congested glomeruli with thickened basement membrane indicative of immune complex deposition (Figure 3b and 3c). In the fluorescence microscopic examination, low fluorescence signal was detected in FITC-labeled kidney sections of rabbits immunized with native LDL (Figure 4a). However, kidney sections of D-ribose-LDL-immunized rabbits showed GBM having a series of intense fluorescence points (Figure 4b and 4c). The deposition of immune complexes in the kidney points toward the possible involvement of D-ribose in glomerulonephritis.

Our research group and others too have shown that D-ribose causes structural perturbations in the LDL molecule [16] resulting in the metabolic abnormalities associated with glycation of LDL molecule that include diminished recognition of LDL by the classic LDL receptor; increased covalent binding of LDL in vessel walls; enhanced uptake of LDL by macrophages, thus stimulating foam cell formation; increased platelet aggregation; generation of neo-antigenic epitopes which are recognized as non-self by the immune system, thus breaking the immune tolerance existing normally to self-antigens. Formation of LDL-immune complexes; and generation of oxygen free radicals, resulting in oxidative damage to both the lipid and protein components of LDL and to any nearby macromolecules. We have also indicated its possible role in glomerulonephritis, an aspect that needs further probe. Our results would pave the way for further studies to evaluate the role of AGE-LDL in the progression and pathophysiology of diabetes-mediated atherosclerosis.

Recently, the presence of auto-antibodies in experimental and diabetes mellitus patients against the glycated DNA was investigated by our group [33]–[36]. There are also couple of recent reports for the presence of the auto-antibodies in the sera of the cancer patients which needs to be validated further [37]. Therefore the need of the hour is to stop this dreaded reaction using novel approach like nanoconjugation [38], [39]. Our research group has recently shown some medicinal plants that have potent antioxidant property like *Phyllanthus virgatus*, *Ficus virens* and *Boerhaavia diffusa* [40]–[42]. Eventually, based on its antioxidant and antidiabetic characteristics, it is hypothesized that these plants might exhibit antiglycating properties as well. This is just a hypothesis to prove the same to get rid of the anomalies happening due to the glycation reaction as discussed above. We hope our findings would also help the way for the treatment of diabetes and its secondary complications like retinopathy, neuropathy and nephropathy.
